# Dissolved molecular hydrogen (H_2_) in Peritoneal Dialysis (PD) solutions preserves mesothelial cells and peritoneal membrane integrity

**DOI:** 10.1186/s12882-017-0741-0

**Published:** 2017-10-31

**Authors:** Masaaki Nakayama, Wan-Jun Zhu, Kimio Watanabe, Ayano Gibo, Ali M. Sherif, Shigeru Kabayama, Sadayoshi Ito

**Affiliations:** 1 Tohoku University, Tohoku University Hospital, Research Division of Chronic Kidney Disease and Dialysis Treatment, 1-1 Seiryo-machi, Aoba-ku, Sendai city, 980-8574 Japan; 2 Tohoku University, United Centers for Advanced Research and Translational Medicine, Center for Advanced and Integrated Renal Science, Sendai, Japan; 3 Fukushima Medical University, Fukushima, Japan; 4 Trim Medical Institute Co., Ltd., Osaka, Japan; 5 The Tokyo Jikei University School of Medicine, Department of Nephrology and Hypertension, Tokyo, Japan

**Keywords:** Molecular hydrogen, Electrolyzed water, Biocompatibility, PD solution, Mesothelial cell, Macrophage

## Abstract

**Background:**

Peritoneal dialysis (PD) is used as renal replacement therapy in patients with end-stage kidney disease. However, peritoneal membrane failure remains problematic and constitutes a critical cause of PD discontinuation. Recent studies have revealed the unique biological action of molecular hydrogen (H_2_) as an anti-oxidant, which ameliorates tissue injury. In the present study, we aimed to examine the effects of H_2_ on the peritoneal membrane of experimental PD rats.

**Method:**

Eight-week-old male Sprague-Dawley rats were divided into the following groups (*n* = 8–11 each) receiving different test solutions: control group (no treatment), PD group (commercially available lactate-based neutral 2.5% glucose PD solution), and H_2_PD group (PD solution with dissolved H_2_ at 400 ppb). Furthermore, the influence of iron (FeCl_3_: 5 μM: inducer of oxidative cellular injury) in the respective PD solutions was also examined (Fe-PD and Fe-H_2_PD groups). The H_2_PD solution was manufactured by bathing a PD bag in H_2_-oversaturated water created by electrolysis of the water. Twenty mL of the test solutions were intraperitoneally injected once a day for 10 days. Parietal peritoneum samples and cells collected from the peritoneal surface following treatment with trypsin were subjected to analysis.

**Results:**

In the PD group as compared to controls, a mild but significant sub-mesothelial thickening was observed, with increase in the number of cells in the peritoneal surface tissue that were positive for apoptosis, proliferation and vimentin, as seen by immunostaining. There were significantly fewer of such changes in the H_2_PD group, in which there was a dominant presence of M2 (CD163+) macrophages in the peritoneum. The Fe-PD group showed a significant loss of mesothelial cells with sub-mesothelial thickening, these changes being ameliorated in the Fe-H_2_PD group.

**Conclusion:**

H_2_-dissolved PD solutions could preserve mesothelial cells and peritoneal membrane integrity in PD rats. Clinical application of H_2_ in PD could be a novel strategy for protection of peritoneal tissue during PD treatment.

## Background

Peritoneal dialysis (PD) can be used as home-based therapy for patients with end-stage kidney disease and, worldwide, has played an important role in patient rehabilitation over the last three decades [1, 2]. However, PD is not as robust as hemodialysis with regard to safety and long-term performance. Its Achilles’ heel includes progressive injury to the peritoneal membrane [3, 4] and development of encapsulating peritoneal sclerosis (EPS), which is the most severe complication of PD therapy [5–7].

Mesothelial cell injury is the first step in the development of peritoneal fibrosis, later leading to sclerosis [8, 9], with bio-incompatibility of the PD solution and toxic molecules playing a central role in the pathology [10]. These molecules include glucose and glucose degradation products (GDPs) in the PD solution [11–13], and exogenous oxidants, such as iron [14, 15]. Recent histological studies have reported that the use of neutral PD solutions with low GDPs are beneficial in ameliorating histological changes in the membrane [16, 17], although the risk of EPS remains an issue of serious concern, even in patients treated with neutral PD solutions [18, 19]. This highlights the need for development of more biocompatible PD solutions.

Recent studies have shown dihydrogen (H_2_) has a biological action as an anti-oxidative and anti-inflammatory molecule [20]. H_2_ dissolved in water, given orally or by intraperitoneal administration, can suppress oxidative or inflammatory injury in various types of animal models, by playing a role as modulator of the expression of various molecules, such as MAPK, MEK-1, NFκB, and caspase-3 and 12, and by upregulating Nrf-2, which could prevent oxidative injury and apoptosis [21]. Thus, addition of H_2_ to PD solutions could represent a unique clinical approach to protecting the mesothelial cells and peritoneal tissue of these patients [22, 23].

In the present study, we examined the effects of H_2_ in experimental rats treated with PD solution, to clarify whether adding H_2_ to PD solutions could preserve mesothelial cells and the peritoneal membrane, including in cases of enhanced oxidative injury.

## Methods

### Treatment protocol

Male Sprague-Dawley rats aged 8–10 weeks old were housed under controlled environmental conditions (temperature 22 ± 1.5 °C; humidity 55% ± 5%; 12 hourly dark: light cycle with lights on at 7 a.m.) with free access to water and standard pellet food (0.8% NaCl; Nihon CLEA Japan, Inc., Tokyo, Japan).

All procedures in this study were conducted in accordance with the National Institutes of Health Guide for the Care and Use of Laboratory Animals, and the study protocols were approved by the Animal Committee of Fukushima Medical University (approval number: 25,017).

The rats were divided into three groups: control group (*n* = 9; no treatment), PD group (*n* = 11; treatment with a commercially available 2.5% glucose peritoneal dialysis solution for intraperitoneal use), and the H_2_PD group (*n* = 8; treatment with the same intraperitoneal solution as the PD group, but with addition of 400 ppb of dissolved H_2_). Furthermore, in order to enhance the oxidative stress resulting from the PD solution, the influence of the addition of iron (FeCl_3_: 5 μM) in the PD solution was also examined in another set of rats divided into Fe-PD (*n* = 8) and Fe-H_2_PD groups (*n* = 8).

All groups were treated for ten weekdays, while being fed standard pellet food. Body weight was measured at the beginning and end of the experiment.

Twenty mL of the respective test solutions were intraperitoneally injected into the lower abdomen of rats in the different groups using a 20 gauge needle, once a day for 10 days.

### Hydrogen gas loading into the PD solution and loss after loading

**Fig. 1:**
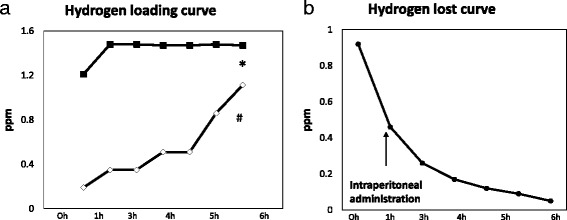
Procedure for producing H_2_-dissolved peritoneal dialysis solution by the bathing method. **a** Time-course of changes in dissolved H_2_ levels in electrolyzed water (*) and the solution in the PD bag (#). **b** Time-course of changes in dissolved H_2_ levels in the solution in the PD bag when placed in air. Water was electrolyzed by Nafion (Synthetic polymer membrane; DuPont, Wilmington, DE) to generate highly dissolved H_2_ water, with the concentration of H_2_ exceeding 1.5 ppm. Then, the PD bag was bathed in the water to allow H_2_ to shift into the PD solution by diffusion (**a**). Dissolved hydrogen is lost rapidly after loading once it is exposed to room air (**b**). Thus, the H_2_-dissolved PD solution was subjected to experiments within 1 h of 12-h bathing, to ensure a high level of H_2_ in the PD solution (>400 ppm)

Hydrogen gas (H_2_) loading into the PD solution was performed by bathing the bag in H_2_-enriched electrolyzed water generated using an electrolyzer (Nihon Trim, Osaka, Japan), as reported elsewhere [22]. Details of the process are shown in Fig. 1.

### Collection of peritoneal tissue and sample analysis

All animals were sacrificed on the final day of the study (15th day). Pentobarbital (50 mg/kg) was administered intraperitoneally for euthanasia. Then, the abdominal cavity was opened and peritoneal tissue samples were carefully collected from the abdominal parietal wall. The samples were fixed in 10% buffered formalin, embedded in paraffin and serially sectioned at a thickness of 2.5 μm. Only the centers of the tissue samples were used for histological analysis, the edges being removed after the samples were fixed in formalin. Rat peritoneal histological examination was conducted under light microscopy with Masson staining and immunohistochemistry staining. Half of each sample was also used for collection of peritoneal cells. For this, peritoneal tissues were soaked in trypsin solution and incubated at 37 °C for 50 min. Then, the suspended cells were treated with Trizol (Thermo Fisher Scientific, Waltham, MA). The extracted nucleic acids were used for real-time polymerase chain reaction (RT-PCR) testing using the two-step RT-PCR kit (Bio-Rad, Hercules, CA) and Agilent array (Takara Bio, Kusatsu, Shiga, Japan).

Immunohistochemical analysis was performed using monoclonal antibodies against the mesenchymal marker vimentin (Santa Cruz Biotechnology Inc., Dallas, TX), proliferative marker Ki-67 (Novus Biologicals, CO), apoptosis marker M30 CytoDeath (PEVIVA, Sundbyberg, Sweden), total macrophage CD68 mouse anti-rat monoclonal (ED1) antibody (LSBio, Seattle, WA), M1 macrophage CD80 mouse monoclonal antibody (Origene Technologies Inc., Rockville, MD), and M2 macrophage CD163 mouse monoclonal antibody (Leica Biosystems, Nussloch, Germany).

For quantitative analysis, the number of positive cells in the peritoneal tissue sample was counted in five randomly selected fields. The positive cells in each picture were counted in relation to per surface length. The surface length of peritoneum was measured by the free software, ImageJ. The average surface length of peritoneum was 220 μm, which corresponds to 9 pixels in the software. We randomly selected 5 fields for image quantification. With regard to analysis of shed cells, the surface length of the peritoneal membrane with/without mesothelial cell covering was measured by ImageJ in 5 randomly taken pictures, and the proportion of uncovered area was calculated by the following formula: (uncovered length/total surface length observed).

RT-PCR analysis was performed using probe sets from the Bio Rad CFX96 system (Bio Rad Laboratories Inc., Hercules, CA). Gene-specific primers for glyceraldehyde-3-phosphate dehydrogenase (GAPDH): forward GGCACAGTCAAGGCTGAGAATG, reverse ATGGTGGTGAAGACGCCAGTA); Vascular endothelial growth factor (VEGF): forward ATCATGCGGATCAAACCTCACC, reverse GGTCTGCATTCACATCTGCTATGC; B-cell lymphoma 2 (BCL2): forward CCTGTGGATGACTGAGTACCTGAAC, reverse CAGAGTCTTCAGAGACAGCCAGGA; Smooth muscle actin A (αSMA): forward GCTCTGTAAGGCGGGCTTTG, reverse ACGAAGGAATAGCCACGCTCA; E-cadherin: forward CAGGAT TACAAGTTCCCGCCA, reverse CACTGTCCGCTG CCTTCA; BCL-2-like protein 4 (BAX): forward CTGCAGAGGATGATTGCTGA, reverse GATCAGCTCGGGCACTTTAG, BCL-2-associated death promoter (BAD): forward CAGTGATCTGCTCCACATTC, reverse TCCAGCTAGGATGATAGGAC; Vimentin: forward AATTGCAGGAGCTGAATGAC, reverse AATGACTGCAGGGTGCTCTC; SNAIL: forward GCTCCTTCCTGGTCAGGAAG, reverse GGCTGAGGTACTCCTTATTAC; and Cytokeratin: forward GAGGAGACCAAAGGCCGTTAC, reverse GAGGAGAATTGAGAGGATGAGGA, were used for amplification of specific complementary (cDNA)s with the iScript one-step RT-PCR kit, also from Bio Rad. The relative expression levels of each messenger RNA (mRNA) were normalized to GAPDH mRNA levels.

Cluster analysis (Pearson’s correlation coefficient) of the results of the Agilent array was performed using a microarray data analysis tool (Filgen Inc., Nagoya, Japan).

### Statistical analysis

Statistical analyses were performed using Sigma plot version 12 (Hulinks, Tokyo, Japan). All results were expressed as the mean ± standard error. Comparisons of groups were performed using one-way ANOVA and the Tukey post-hoc test. Values of *p* < 0.05 were considered to indicate statistical significance.

## Results

### Histological examinations: Masson and immunohistochemistry staining in PD and H_2_PD groups

**Fig. 2:**
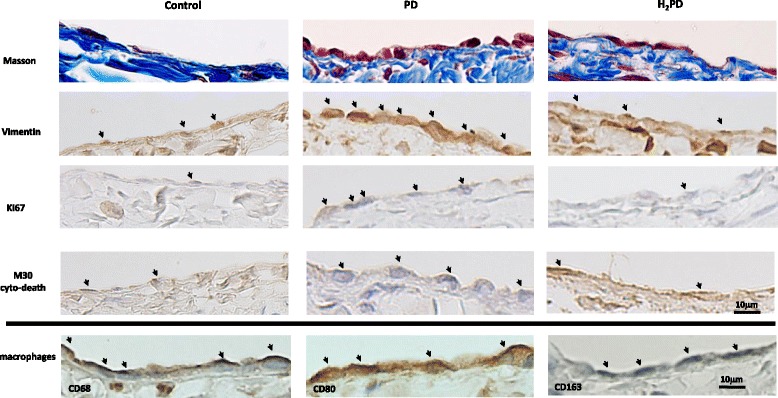
Representative histological findings in the peritoneum: Masson and immunohistochemistry staining in PD and H_2_PD groups. Mesenchymal marker: vimentin; proliferative marker: Ki-67; apoptosis marker: M30 CytoDeath; total macrophage marker: CD68+; M1 macrophage: CD80+; M2 macrophage: CD163+

Representative findings of histological examinations are shown in Fig. 2. Mesothelial cells and sub-mesothelial layers were observed by Masson staining. As compared to the control group, PD group rat peritoneum showed cuboidal changes in the cells on the surface of the membrane, with mononuclear cells infiltrating the surface as well as in the sub-mesothelial layer. The H_2_PD group showed relatively flatter cells on the surface. In terms of immunohistochemical staining, such as for vimentin, Ki-67, and M30 CytoDeath, positive cells were mainly observed in the surface of the peritoneum.

**Fig. 3:**
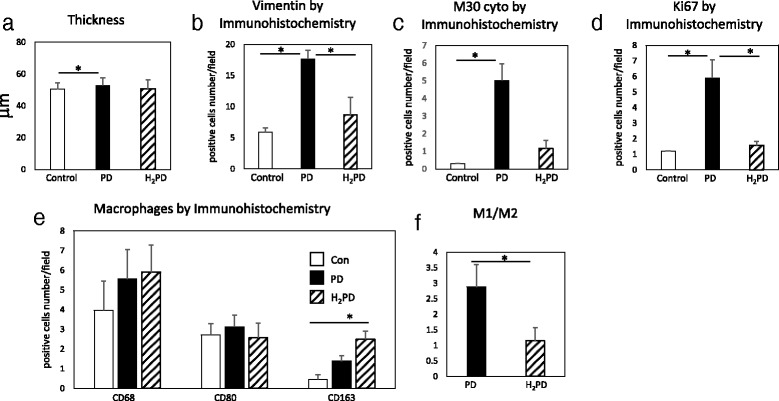
Quantitative analysis of peritoneal thickness and immunohistochemical staining of the peritoneum in PD and H_2_PD groups. Peritoneal thickness (**a**), and immunostainings of mesenchymal marker: vimentin (**b**), proliferative marker: Ki-67 (**c**), apoptosis marker: M30 CytoDeath (**d**), and total macrophage marker: CD68+, M1 macrophage: CD80+, and M2 macrophage: CD163+ (**e**), and ratio of M1 /M2 (positive cells number per field) (**f**). * *p* < 0.05

There were significant differences in the thickness of the sub-mesothelial layer among the three groups (46.6 ± 2.6 μm in the control, 52.9 ± 4.6 in the PD, and 50.7 ± 5.6 in the H_2_PD group; control vs. PD: *p* < 0.05) (Fig. 3a).

A significant increase in the number of vimentin-positive cells were observed in the PD group compared with the control and H_2_PD groups (5.9 ± 0.6 positive cells/field in the control, 17.7 ± 1.4 in the PD, and 8.7 ± 2.8 in the H_2_PD group; PD vs. control and H_2_PD groups: *p* < 0.05, respectively) (Fig. 3b).

Ki-67-positive cells were significantly increased in the PD group as compared with the control and H_2_PD groups (1.2 ± 0.0 positive cells /field in the control, 5.9 ± 1.2 in the PD, and 1.6 ± 0.3 in the H_2_PD group; PD vs. control and H_2_PD groups: *p* < 0.05, respectively) (Fig. 3c).

M30 CytoDeath-positive staining was significantly increased in the PD group as compared with the control and H_2_PD groups (0.3 ± 0.0 positive cells/field in the control, 5.0 ± 1.0 in the PD, and 1.2 ± 0.5 in the H_2_PD group: PD vs. control and H_2_PD groups: *p* < 0.05, respectively) (Fig. 3d).

There were no significant differences in total macrophage infiltration (C68), and M1 macrophages (CD80) in the peritoneum among the three groups. However, there was a significant difference in the infiltration of M2 macrophages (C163) (0.4 ± 0.1 positive cells/field in the control, 1.4 ± 0.3 in the PD, and 2.2 ± 1.2 in the H_2_PD group; control vs. H_2_PD: p < 0.05) (Fig. 3e). There were statistically significant differences in the ratios of M1/M2 macrophage infiltration in the peritoneum between the PD and H_2_PD groups (2.9 ± 0.7 in the PD and 1.1 ± 0.4 in the H_2_PD group, p < 0.05) (Fig. 3f).

### Real-time PCR in PD and H_2_PD groups

**Fig. 4:**
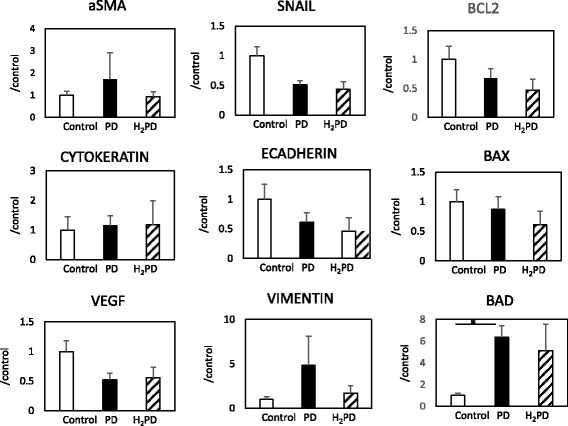
Results of real-time PCR of peritoneal cells in PD and H_2_PD groups. * *p* < 0.05 vs. control VEGF: Vascular endothelial growth factor, BCL2: B-cell lymphoma 2, αSMA: Smooth muscle actin A, BAX: BCL-2-like protein 4, BAD: BCL-2-associated death promoter

In the PD group, there was a significant increase in gene expression of BAD as compared with the control group. There were tendencies for increased expression of genes, such as aSMA and vimentin, in the PD group as compared with the other groups, although the differences between them were not statistically significant (Fig. 4).

### Agilent array in PD and H_2_PD groups

**Fig. 5:**
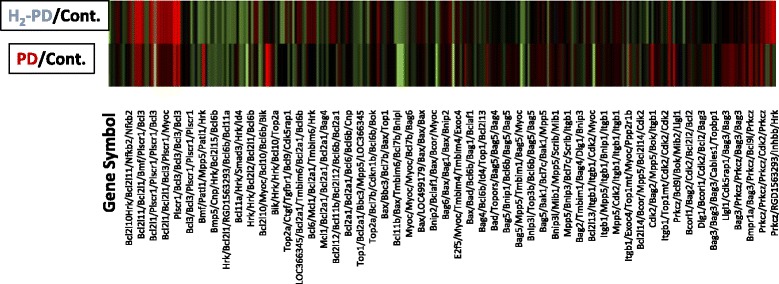
Representative DNA Agilent array of peritoneal cells. Relative differences in representative gene expressions between H_2_PD and PD groups

Whole gene expression, performed using a Microarray data analysis tool, indicated that there were differences in total gene expression levels by 8.7% between PD and control groups, and by 3.7% between H_2_PD and PD groups, respectively (Fig. 5).

### Comparisons of Fe-PD and Fe-H_2_PD groups

**Fig. 6:**
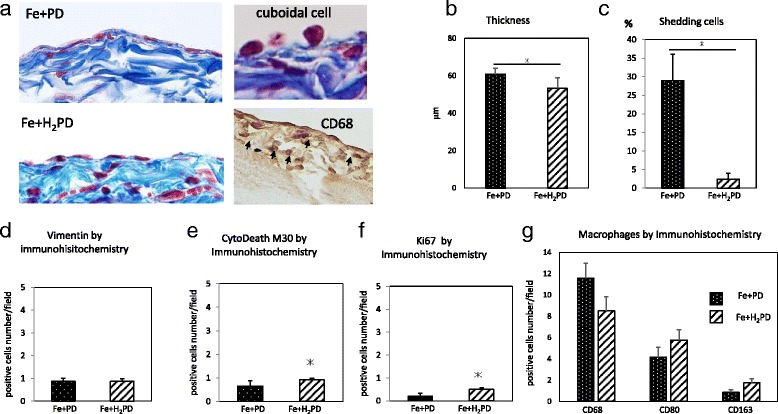
Comparison of Fe-PD and Fe-H_2_PD groups. Representative findings of Masson and immunohistochemical staining (CD68) (**a**), and quantitative analysis of peritoneal morphology and immunohistochemical staining of the peritoneum in the respective groups (**b**-**g**) are shown. Peritoneal thickness (**b**), ratio of shed cells in the peritoneal surface (**c**), immunostainings of mesenchymal marker: vimentin (**d**), apoptosis marker: M30 CytoDeath (**e**), proliferative marker: Ki-67 (**f**), and total macrophage marker: CD68+, M1 macrophage: CD80+, and M2 macrophage: CD163+ (**e**), respectively. * *p* < 0.05

Representative findings of Masson and immunohistochemical staining in the Fe-PD and Fe-H_2_PD groups are shown in Fig. 6a. In the Fe-PD group, severe shedding of mesothelial cells was observed, and the cells remaining on the peritoneal surface included a mix of cuboid and aggregated cells. The findings were similar in the Fe-H_2_PD group, although the extent of shedding was less than in the Fe-PD group.

Significant differences were found in the thickness of the sub-mesothelial layer between the groups (60.9 ± 3.1 μm in the Fe-PD, and 53.3 ± 5.7 in the Fe-H_2_PD group; *p* < 0.05) (Fig. 6b), and the proportion of shed cells on the peritoneal surface (28.9 ± 7.1% in the Fe-PD, and 2.4 ± 1.6% in the Fe-H_2_PD group; *p* < 0.05) (Fig. 6c). There were no significant differences in vimentin-positive cells (Fig. 6d), although there were statistically significant differences in M30 CytoDeath-positive cells (0.7 ± 0.1 and 0.9 ± 0.2 positive cells/field in the Fe-HD and Fe-H_2_PD groups, respectively; *p* < 0.05) (Fig. 6e), and in Ki-67-positive cells (0.2 ± 0.1 and 0.5 ± 0.1 positive cells/field in the Fe-PD and Fe-H_2_PD groups, respectively; *p* < 0.05) between the groups (Figs. 6f).

There were no significant differences in the infiltration of all types of macrophages (C68), M1 macrophages (CD80), and M2 macrophages (CD163) in the peritoneum between the two groups (Fig. 6g).

## Discussion

In the present study, we examined the potential of an H_2_-containing PD solution (400 ppb) in the protection of peritoneal mesothelial cells and peritoneal tissue in experimental PD rats. We employed a commercially available low-GDP, neutral PD solution for the study, and also studied a solution with FeCl_3_, to enhance oxidative stress-induced tissue injury.

We observed mild but significant sub-mesothelial thickening in the PD group as compared to controls. Notably, analysis of the PD group indicated characteristic morphological changes in the mesothelial cells, in the form of cuboidal formation and nuclear aggregation. Further, there was increased immunostaining suggestive of apoptosis, proliferation, and vimentin in the peritoneal surface tissue. On the other hand, there were significantly fewer changes in the H_2_PD group, which instead exhibited a dominant presence of M2 type macrophages. The Fe-PD group exhibited significant loss of mesothelial cells and sub-mesothelial thickening, although these changes were ameliorated in the Fe-H_2_PD group.

In this study, there were unique findings in the PD group, namely simultaneous increases in proliferation and apoptosis in the surface cells. Although the exact mechanism of this finding remains to be elucidated, we suggest the following hypothesis: the mesothelial cells are constantly exposed to PD solution, which is potentially bio-incompatible. The mesothelial cells seem to be in both a damaged “pre-apoptotic” state and in a state of compensatory proliferation in order to restore the membrane. The balance between the two opposite states is probably crucial for preservation of peritoneal integrity. In fact, the disruption in balance caused by oxidative stress secondary to FeCl_3_ resulted in severe mesothelial loss, along with an increase in accompanying membrane thickness (Fig. 6b and c).

Of note in the present study, morphological changes were found in the surface cells of the peritoneum in the PD group, in the form of cuboidal changes in the cells. There was a significant increase in vimentin staining in the PD group, as compared to the control and H_2_PD groups, which may indicate phenotypic alteration of mesothelial cells, resulting in epithelial-mesenchymal transition (EMT). However, unexpectedly, the expression of genes that modulate EMT was not different between the PD and H_2_PD groups. Hence, it remains unclear whether the same or different mesothelial cells presented with apoptosis and proliferation. This needs to be addressed in future studies.

With regard to PCR analysis, we chose EMT and its related genes, and anti-apoptotic and apoptotic genes, because we originally hypothesized that H_2_ could ameliorate activation of the process of EMT and the oxidative cellular injury resulting from exposure to PD solutions. We expected differences in gene expressions between the PD and H_2_PD groups, e.g. increases in SNAIL, vimentin, aSMA and VEGF, and decreases in ECADHERIN and CYTOKERATIN in the former group, and increases in BAX and BAD, and decreases in BCL2 in the latter group. However, unexpectedly, there were no differences in gene expressions between the two groups (Fig. 4). Therefore, we suppose other potential mechanisms for the effect of H_2_ on membrane protection.

Recent studies have revealed a significant role of tissue macrophages in orchestrating the healing process in wound tissue, i.e., M1 macrophages have inflammatory actions, and M2 have remodeling/healing actions in damaged tissues [24]. In this study, there were significant differences in infiltration of M2 macrophage sub-populations in the peritoneum between the PD and H_2_PD groups, with M2 being dominant in the H_2_PD group. This may indicate enhanced healing of peritoneal tissue in the H_2_PD group. However, the question as to whether H_2_ can facilitate macrophage functional switching in order to reduce tissue injury needs to be clarified in future.

Improved biocompatibility of PD solutions is the most crucial factor in preserving peritoneal integrity and ensuring successful long-term PD. Although low-GDP neutral PD solutions are supposedly biocompatible, as compared to conventional acidic solutions with high GDPs [16, 17], the present results indicate that the neutral solution is still somewhat bio-incompatible. Cluster analysis, which revealed an 8.7% difference in gene expression profiles between the PD and control groups, may well support this notion. We suppose that high glucose may, at least partly, play a crucial role as an oxidant, as reported elsewhere [11–13], which indicates room for improvement in the biocompatibility of the present standard solutions. Studies on clinical approaches to peritoneal protection have been limited so far [25–27].

To date, no critical adverse effects of H_2_ have been reported in humans, making it seem like a very good candidate for clinical application, provided there is scientific rationale for its use. We previously produced an H_2_-dissolved hemodialysis solution and observed an improvement in hypertension, as well as decreases in plasma levels of MCP-1 and MPO in chronic hemodialysis patients [28–30]. We believe that H_2_-containing PD solutions could be a candidate as novel PD solutions with improved biocompatibility, and our results support the significance of H_2_PD clinical trials in the future. In future clinical trials, the risks of flammable H_2_ gas need to be taken into account. However, the explosive concentration of H_2_ is close to 4% (40 × 10^3^ ppm), while the levels of H_2_ in PD solution bags are less than 0.4 ppm, and H_2_ levels of water for bathing manufactured by the present method are 1.6 ppm at maximum (Fig. 1a). Therefore, we believe it is safe to conduct clinical trials using this system.

## Conclusion

H_2_-dissolved PD solutions could preserve mesothelial cells and the peritoneal membrane in experimental PD rats. Clinical application of H_2_ in PD could lead to creation of a novel strategy for the protection of peritoneal tissues during PD treatment.

## Abbreviations

CD163+: M2
CD80 +: M1
ED1, CD68: Macrophages
EMT: epithelial mesenchymal transition
EPS: encapsulating peritoneal sclerosis
GDP: glucose degradation products
H_2_: dihydrogen, molecular hydrogen
MAPK: mitogen-activated protein kinase
MCP-1: monocyte chemotactic and activating factor
MEK: MAPK/ERK kinase
MPO: myeloperoxidase
NFkB: nuclear factor-kappa B
Nrf2: NF-E2 related factor 2
PD: Peritoneal dialysis
Ppb: parts per billion
Ppm: parts per million
